# A Book on Cancer
*The Cure of Cancer and How Surgery Blocks the Way. By Jno. Shaw, M.D.Lond. F. S. Turney, London, 1907. Pp. xx. and 229.


**Published:** 1907-07-06

**Authors:** 


### A BOOK ON CANCER.*

The book with which we have here to deal is written by Dr. Jno. Shaw. We may say at once that it is a most disappointing production. Its title is charged with great expectations, but the measure of its achievement is limited in the extreme. Of loose, vague, prolix writing there is an abundant supply, but definite and practical evidence is indeed sadly to seek.

### Its Main Theses.

The main theses of the book are two in number. First, that cancer can be cured without operation. Secondly, that increased operative activity is responsible for an increased cancer mortality. These are simple and direct propositions. They are also propositions of great moment. It might, therefore, have been expected that the writer who advances them would have addressed himself to a serious presentation of the evidence necessary for their support. But such is not the method of Dr. Shaw. If the position he adopts is well founded, he is in possession of a form, or forms, of treatment by means of which malignant disease can be successfully treated. Yet his book may be read from cover to cover without the reader gaining any direction as to what should be done in, say, a plain and manifest case of carcinoma of the breast. Should such a case be dealt with by the surgeon? Or should the patient be told that removal of the breast is unnecessary? And if the latter, what form of treatment should be advised? These surely are the questions to which answers might be anticipated in a book professing to deal with the cure of cancer.

But in Dr. Shaw's book we look for such answers in vain. It is true that the author's attitude is distinguished by a wholesale distrust—not always very courteously expressed—of both the practitioners and the procedures of surgery. It is also true that he names a long list of agents which at various times have been claimed as remedies in the treatment of cancer. But of the great majority of these he appears to have had no personal experience, and, in the end, we are left utterly in the dark whether in any individual case of cancer we ought to prescribe compression, galvanism, *x*-rays, high-frequency currents, arsenic, iodine, bromine, iron, conium, opium, antipyrine, belladonna, mezereon, chian turpentine, eucalyptus oil, cinnamate of sodium, violet leaves, ox-gall, soap solution, trypsin, iodipin, potassium iodide, thyroid gland, intestinal gland, mammary gland, lymphatic gland, or "sandwiches of raw-heart muscle." It is nothing to the point to quote individual instances in which writers of more or less authority have claimed good results from the use of one or other of these various substances. Such references are familiar to every practitioner, as are also the occasional records of the spontaneous disappearance of apparently malignant growths.

The question is not, Do such things occur? but, Are they a valid basis for practice? To make such exceptional experiences the opportunity for informing patients, as Dr. Shaw does on his first page, that "cancer is recoverable to the very uttermost" seems to us as morally indefensible as it is illogical. At least this is the verdict which must be passed on such a statement in the absence of a plain indication of the methods by which recovery is to be assured.

Instead of addressing himself to the essential issue suggested by the title of his book —an issue of momentous and immediate importance to thousands of doomed and despairing men and women—Dr. Shaw occupies himself with such trifles as the history of cinchona bark and the Bradshaw lecturer's infelicitous quotations, and fills his pages with musty paragraphs from medical literature, the majority of which have little concern with the urgent question he professes his ability to answer. Hence, in spite of his heroics and moralisings, we cannot recognise that he has any adequate appreciation of the responsibility he has assumed. Men through long years have waited wearily and with hope deferred for a knowledge of the successful treatment of cancer. And yet, in face of this, and in the presence of a multitude of unhappy victims, Dr. Shaw has considered it seemly to deal with the subject in the way we have described. We do not envy him the terrible responsibility he has incurred.

### Its Conclusion.

As for the contention that to surgery is due an increase in the cancer mortality, the principal argument advanced is that surgical operations are more numerous now than was formerly the case, and that the same is true of deaths from cancer. With this kind of logic Dr. Shaw is presumably content. We make no question of his earnestness and sincerity; but he must not expect his readers to be so easily satisfied. But we do say that his book shows him to be wholly wanting in a recognition of the serious and solemn responsibility assumed by any medical practitioner who professes the possession of knowledge by which cancer can be cured, and who at the same time abstains from a clear definition of the methods by which this end is to be attained. Dr. Shaw has complained that certain opportunities for publicity have been denied him. The complaint is absurd, for there is no one the medical profession and the public will hear more gladly than the fortunate discoverer of a successful method of relieving and curing the victims of malignant disease. In his own book, at all events, he has a full opportunity, and as we must assume that he has now delivered his soul, we can only regret that the result is so ridiculously unequal to the claims advanced in its favour. So far as our influence extends, we emphatically protest against the attempt to claim for this thing of shreds and patches, of puerilities, and secondhand gossip, the distinction of a serious work on the cure of cancer.

* The Cure of Cancer and How Surgery Blocks the Way. By Jno. Shaw, M.D.Lond. F. S. Turney, London, 1907. Pp. xx. and 229.

